# RETRACTION: Obesity, Insulin Resistance, and Metabolic Syndrome: A Study in WNIN/Ob Rats from a Pancreatic Perspective

**DOI:** 10.1155/bmri/9761801

**Published:** 2026-03-26

**Authors:** BioMed Research International

RETRACTION: V. Venkatesan, S. L. Madhira, V. M. Malakapalli, M. Chalasani, S. N. Shaik, V. Seshadri, V. Kodavalla, R. R. Bhonde, G. Nappanveettil, “Obesity, Insulin Resistance, and Metabolic Syndrome: A Study in WNIN/Ob Rats from a Pancreatic Perspective,” *BioMed Research International*, 2013, 617569, https://doi.org/10.1155/2013/617569

The above article, published online on 15 December 2013 in Wiley Online Library (wileyonlinelibrary.com), has been retracted by agreement between the journal Editor‐in‐Chief, Yoel Klug and John Wiley & Sons Ltd. The retraction has been agreed due to multiple errors identified in the figures, initially on PubPeer [1].

• The Isotype control/DAPI panel in Figure 2e appears identical to the image shown in Figure 3c
• The 1 month/Lean panel and the 6 months/mutant panel shown in Figure 3b appear to share overlapping, repeated elements
• The 1 month/control and 1 month/mutant panels shown in Figure 5a appear to share overlapping, repeated elements
• There appears to be a duplication between the Figure 3e and Figure 7e panels

After assessment of the author’s response by the Chief Editor, the results and conclusions are deemed unreliable and the article is therefore retracted.

The authors disagree with the retraction.
